# fMRI Scanner Noise Interaction with Affective Neural Processes

**DOI:** 10.1371/journal.pone.0080564

**Published:** 2013-11-18

**Authors:** Stavros Skouras, Marcus Gray, Hugo Critchley, Stefan Koelsch

**Affiliations:** 1 Cluster of Excellence “Languages of Emotion”, Freie Universität Berlin, Berlin, Germany; 2 Clinical Imaging Sciences Centre, Brighton and Sussex Medical School, Brighton, East Sussex, United Kingdom; 3 Department of Psychology, University of Sussex, Falmer, East Sussex, United Kingdom; 4 Centre for Advanced Imaging, University of Queensland, St Lucia, Queensland, Australia; 5 Sackler Centre for Consciousness Science, University of Sussex, East Sussex, United Kingdom; University Medical Center Goettingen, Germany

## Abstract

The purpose of the present study was the investigation of interaction effects between functional MRI scanner noise and affective neural processes. Stimuli comprised of psychoacoustically balanced musical pieces, expressing three different emotions (fear, neutral, joy). Participants (N=34, 19 female) were split into two groups, one subjected to continuous scanning and another subjected to sparse temporal scanning that features decreased scanner noise. Tests for interaction effects between scanning group (sparse/quieter vs continuous/noisier) and emotion (fear, neutral, joy) were performed. Results revealed interactions between the affective expression of stimuli and scanning group localized in bilateral auditory cortex, insula and visual cortex (calcarine sulcus). Post-hoc comparisons revealed that during sparse scanning, but not during continuous scanning, BOLD signals were significantly stronger for joy than for fear, as well as stronger for fear than for neutral in bilateral auditory cortex. During continuous scanning, but not during sparse scanning, BOLD signals were significantly stronger for joy than for neutral in the left auditory cortex and for joy than for fear in the calcarine sulcus. To the authors' knowledge, this is the first study to show a statistical interaction effect between scanner noise and affective processes and extends evidence suggesting scanner noise to be an important factor in functional MRI research that can affect and distort affective brain processes.

## Introduction

 The increasing interest of the neuroscientific community in applying research findings towards the development of clinical applications to complement diagnostic, therapeutic and surgical praxis makes the issue of scanner noise particularly pressing. An extensive review [1] highlighted that the interference of scanner noise on normal brain function can be particularly pronounced during cognitive task performance in neurodegenerative and psychopathological populations, which have greater difficulty in attending to task-related stimuli [2,3]. Moreover, interference from scanner noise is especially important in the case of functional MRI (fMRI) utilization for planning of surgical removal of brain tissue, due to the need for accurate functional mapping and the influence of scanner noise on brain activation patterns [4-7]. Functional mapping is complicated by the fact that when improving resolution, by means of increasing the strength of the magnetic field utilized during imaging, the intensity of scanner noise also increases, thereby compromising progress in resolution by the increased noise interference [8,9]. 

 Scanner noise is intrinsically linked to the usual scanning implementation. In brief, magnetic resonance tomographers create strong momentary magnetic fields which cause the axes of the atoms of the objects being scanned to align momentarily and then return to their original orientation, emitting electromagnetic energy that comprises the fMRI datum [10]. The rapid changes of the magnetic forces cause magnetic elements of the apparatus to expand and contract in fast frequencies, resulting in a repetitive audibly loud sound, the scanner noise, which compromises the conditions of measurement [11] and constitutes a disadvantage of fMRI compared to positron emission tomography, magnetoencephalography and electroencephalography. In principle, the scanner noise is acting as a nuisance stimulus with undesirable effects, such as raising the baseline neural activity in the auditory cortex [12,13], thereby decreasing the already low signal-to-noise ratio and percent signal change that is due to experimental manipulation [1,14]. Moreover, scanner noise overlaps in acoustic frequency with certain stimuli [8,15,16], leading to masking effects [17] and an increase in attentional resources required for the disambiguation of signal sources [18-20], as well as nonlinear interactions in the auditory cortex [16,21]. Scanning protocols that feature a decrease in scanner noise, such as sparse temporal sampling [22-24] have been proven to decrease the deleterious effects of scanner noise as demonstrated with fMRI (for reviews see 11,14), magnetoencephalography [25], positron emission tomography [26], as well as electroencephalography experimental results which show that during auditory working memory tasks, listening to recordings of scanner noise can differentially alter significantly the amplitude or latency of the P1, N1, N2 and P3 event related potential components [27]. 

 The deleterious effects of scanner noise were considered by most researchers to exert an influence solely on auditory processes treated as being cognitively separable from most experimental tasks, even though numerous studies suggest that the visual and motor cortices can also be influenced [19,20,26,28,29] and that scanner noise can influence attention as well as memory performance [27,30]. The way effects of scanner noise are modulated in the face of stimuli evoking basic emotions of biological importance, such as joy and fear [31], is not known. 

 Considering the stimuli and methods used in previous studies of scanner noise effects, it is apparent that beyond the consistent impact of scanner noise on the response of the auditory cortex during auditory tasks (reduced percentage of fMRI signal change), there are indications and concerns for more complex scanner noise effects on visual cortex activity [20]. The nature of such effects seems to be related to the task performed, with decreases in activity due to scanner noise during simple sensory stimulation [19,20,28,29] and an increase in activity due to scanner noise during more elaborate cognitive tasks involving mental imagery [26]. The visual, motor and auditory cortices are brain regions where effects can manifest due to emotional factors [32-38]. Because no measure of valence, arousal nor intensity of emotional experiences was obtained during any of the previous studies on scanner noise effects, one cannot preclude the possibility that differences were due to affective factors that were uncontrolled within and across studies. 

 A recent study [39] used consonant (pleasant) and dissonant (unpleasant) versions of 10 second-long musical excerpts, showing that decreasing scanner noise when contrasting responses between pleasant and unpleasant music leads to enhanced detectability of activity in limbic structures related to affective processing. That study reported results from three experiments concerning the sensitivity of continuous temporal scanning, sparse temporal scanning and interleaved silent steady state (ISSS) scanning techniques. ISSS scanning showed stronger activation than sparse temporal scanning, and sparse temporal scanning showed stronger activation than continuous temporal scanning in auditory cortex, amygdala and hippocampal formation. However, no statistical comparison was made between the data obtained using the three different acquisition schemes. Therefore, no previous study has explicitly computed statistical interaction effects between scanner noise and emotion. 

 The present study aims to investigate the possible existence of brain regions where the pattern of affective responses can be altered or even reversed due to scanner noise. Understanding the brain mechanisms implementing emotional experiences is important because emotions seem to be related, in part, to brain processes that are particularly vulnerable to stress [40-43], and impaired emotional functioning can be seen as a marker of most psychopathologies [44-48]. Moreover, behavioral interventions that target the emotional constituents of cognition can be successful in alleviating depressive symptomatology [49], and reduction of emotional stress has beneficial effects on immune system function [50-53]. Musical stimulation, being emotional and acoustic, provides a well-suited medium for the investigation of interference by an acoustic distractor, such as scanner noise, on affective processing. 

 To address the question of possible interactions between emotion and scanner noise, a stimulus set was utilized, consisting of fearful, neutral and joyous musical pieces. An independent previous study using the same stimuli [38] showed that the auditory cortex and amygdala, which are the brain regions with the most significant difference in activity between the extremes of this biologically relevant affective dimension, exhibit an incremental increase in activity from fear to joy. The present study tests for an interaction effect between the two-level factor temporal scanning protocol used (sparse vs continuous, that corresponds to reduced vs constant scanner noise, respectively) and the three-level linear factor emotion (fear, neutral, joy). 

 It was hypothesized that the auditory cortex would show an interaction effect because it has been found to be most responsive to such acoustic emotional stimulus sets [37,39], and to be susceptible to acoustic interference due to scanner noise in all related published studies (for reviews see 11,14). Moreover, the present study tested whether the visual and motor cortices would also show interaction effects due to emotional factors that had not been accounted for across previous studies. The study also tested whether limbic structures such as the amygdala and hippocampal formation are influenced by scanner noise. Given the large variety of areas contributing to emotional phenomena, a whole-brain approach was employed. 

## Methods

### Ethics Statement

All subjects gave written informed consent. The study was conducted according to the Declaration of Helsinki and approved by the ethics committee of the School of Life Sciences and the Psychology Department of the University of Sussex.

### Participants

34 individuals (aged 19 - 36 years, M = 23.78, SD = 4.98, 19 females) took part in the experiment. All participants had normal hearing (as assessed with standard pure tone audiometry) and were right-handed (according to self-report). None of the participants was a professional musician or a music student; 19 participants had no or only minimal formal musical training and 15 participants were amateur musicians who had learned at least one musical instrument (mean duration of formal training was 2.6 years). The participants were split into two groups of 17 subjects each; a group that underwent sparse temporal scanning (aged 19 - 36 years, M = 24.53, SD = 6.28, 9 females, mean duration of formal musical training 3.19 years, SD = 3.35) and a group that underwent continuous scanning (aged 19 - 28 years, M = 22.92, SD = 2.90, 10 females, mean duration of formal musical training 1.94 years, SD = 2.28). Independent samples t-tests showed that the two groups did not differ significantly with regards to age and formal musical training (p > 0.05). Exclusion criteria were left-handedness, professional musicianship, a score on Beck’s Depression Inventory [54] of 13 or more points, consumption of alcohol or caffeine exceeding one liter during the 24 hours prior to testing, poor sleep during the previous night, past diagnosis of a neurological or psychiatric disorder, and abnormal brain anatomy. 

### Stimuli

Musical stimuli were selected from CD recordings to evoke joy or fear (see Table S3). Neutral pieces, evoking neither joy nor fear were composed through the use of an algorithm that generated random tones belonging to a pentatonic scale, constrained by prespecified parameters (for more details see 38). There were 8 stimuli per category. Behavioral data (see Results for details) showed that musical stimuli evoked the desired feelings in the sample studied. All stimuli were matched across conditions in triplets (joy, neutral, fear) with regard to tempo (beats per minute), mean fundamental frequency pitch, fundamental frequency pitch variation, pitch centroid value, spectral complexity, and spectral flux. This was confirmed by an acoustic analysis of the stimuli using “Essentia”, an in-house library for extracting audio and music features from audio files (http://mtg.upf.edu/technologies/essentia). The Essentia software was also used to test for differences between stimuli with regard to another 177 acoustical factors. Ten psychoacoustic factors were found to differ significantly between experimental conditions (p < 0.001, corrected for multiple comparisons). These factors were mean and variance of fundamental frequency salience, mean and variance of sensory dissonance, mean chord strength and mean key strength, mean and variance of spectral flux, mean spectral crest and mean spectral complexity (for more details see 38). The values of these factors associated with each stimulus were used in the fMRI data analysis as additional regressors of the general linear model’s design matrix (see Data Analysis for details). 

### Procedure

**Figure 1:**
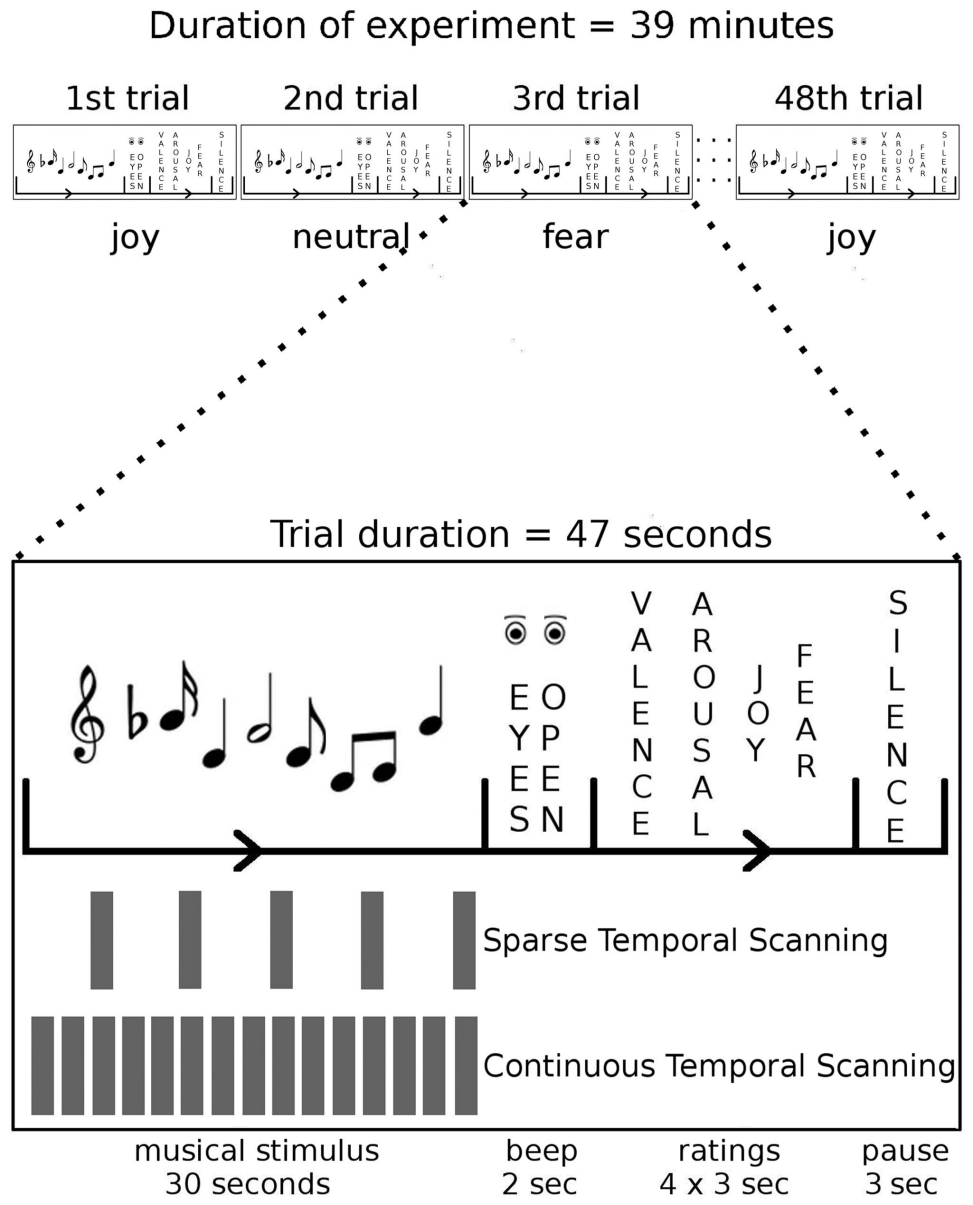
Experimental design. In each trial, a music stimulus (either joy, fear, or neutral) was presented in pseudorandom order for 30 s. Participants listened to the music with their eyes closed. Then, a beep tone signaled to open the eyes and to commence the rating procedure. Four ratings (felt valence, arousal, joy, and fear) were obtained in 12 s, followed by a few seconds of pause. Trial duration was approximately 47 s, the experiment comprised of 48 trials. Grey bars indicate volume acquisitions for sparse and continuous scanning groups (TR = 2 s).

Prior to the MRI session, participants were presented with short (12 s) versions of each stimulus to obtain familiarity ratings: Participants rated their familiarity with each piece on a four-point scale (ranging from “To my knowledge I have never heard this piece before”, to “I know this piece, and I know who composed, or performed it”). One outlier participant, who misinterpreted the familiarity rating scale, was not considered in the analysis of familiarity ratings. Following the familiarity ratings, participants were trained on the emotion rating procedure, using 12 s long excerpts of musical pieces that did not belong to the stimulus set used in the fMRI scanning session. During the fMRI scanning session, stimuli were presented in a pseudo-random order so that no more than two stimuli of each stimulus category (joy, fear, neutral) followed each other. The task of the participants was to listen to the musical stimuli with their eyes closed and to rate their emotional state after each musical stimulus. Each musical stimulus was followed by an interval of 2 s in which a beep tone of 350 Hz and 1 s duration signaled participants to open their eyes and to commence the rating procedure. During the rating procedure, participants indicated how they felt at the end of each excerpt with regard to valence (“pleasantness”), “arousal”, “joy” and “fear”. That is, participants rated how they felt, and not which emotion each song was supposed to express (for the importance of this see 55). Ratings were obtained with 6-point Likert scales (ranging from “not at all” to “very much”). The time interval for the rating procedure was 12 s and each rating period was followed by approximately 3 s of rest (see Figure 1). The entire stimulus set was presented twice during the fMRI scanning session. Musical stimuli were presented using Presentation (version 13.0, Neurobehavioral systems, Albany, CA, USA) via MRI compatible headphones (under which participants wore earplugs). Instructions and rating screens were delivered through MRI compatible liquid crystal display goggles (Resonance Technology Inc., Northridge, CA, USA). 

### MR Scanning

Images were acquired using a 1.5 Tesla scanner (Magnetom Avanto, Siemens AG, Erlangen, Germany) equipped with a standard 12-channel head coil. Prior to the fMRI measurements, a high-resolution (1x1x1 mm) T1-weighted anatomical reference image was acquired from each participant using a rapid acquisition gradient echo sequence. During the fMRI measurements, the continuous scanning group (N=17) underwent Echo Planar Imaging with an echo time of 30 ms and a repetition time of 2 seconds. Slice-acquisition was interleaved within the repetition time interval. The matrix acquired was 64x64 voxels with a Field Of View of 192 mm, resulting in an in-plane resolution of 3 mm. Slice thickness was 3 mm with an interslice gap of 0.6 mm (37 slices, whole brain coverage). The acquisition window was tilted at an angle of 30 degrees relative to the line between the anterior and posterior commissures to minimize susceptibility artifacts in the orbitofrontal cortex [56-58]. All scanning parameters were identical for the sparse scanning group (N=17) apart from the introduction of a 4 s delay between volume acquisitions. 

### Data Analysis

fMRI data were processed using LIPSIA 2.1 [59]. Data were corrected for slicetime acquisition and normalized into MNI-space-registered images with isotropic voxels of 3 cubic millimeters. A temporal highpass filter with a cutoff frequency of 1/90 Hz was applied to remove low frequency drifts in the fMRI time series, and a spatial smoothing was performed using a 3D Gaussian kernel and a filter size of 6 mm Full Width at Half Maximum. A mixed effects block design general linear model analysis was employed [60]. Valence ratings, arousal ratings, familiarity ratings, ten important psychoacoustic parameters (see Stimuli) and realignment parameters were entered in the design matrix as covariates of no interest [61]. On the first level, parametric contrasts were calculated to show brain regions where activity correlates with increases/decreases along the emotional dimension from fear to joy. Two sample t-tests were utilized at the second level to compare the contrast images from the first level between the two groups, computing the interaction effects between the parametric factor emotion (with levels fear, neutral, joy) and scanning group (with levels sparse/quieter and continuous/noisier). 

To clarify the nature of the interaction effects between scanning group and emotion, z−maps were computed and examined for each scanning group separately, for each combination of stimulus categories (i.e. joy vs fear, joy vs neutral, fear vs neutral). The conjunction of each z-map with the z-map of the interaction effect was also computed based on the absolute z-values. Additionally, to certify that interaction effects in the auditory cortex are due to scanner noise modifying affective responses rather than modifying psychoacoustic processes, the same procedure was used to compute z−maps of interaction effects between scanning group and each of the ten important psychoacoustic parameters (see Stimuli). 

All findings were corrected for multiple comparisons by the use of cluster-size and cluster-value thresholds obtained by Monte Carlo simulations with a significance level of p < 0.05. The voxel-wise threshold before applying the Monte Carlo simulation was z=3.09 (corresponding to a probability of 0.001). Note that z=3.09 defined the initial cluster threshold of a randomly generated map of z-values. The LIPSIA multiple-comparisons correction-algorithm then filled the space of brain voxels with randomly generated values and counted the number of false positives. This resulted in pairs of possible thresholds corresponding to cluster size and maximal z-value per cluster. Through this method, the cluster size can be very small if the associated z-value is very large. The derived information regarding possible combinations of cluster-size and z-values was then utilized by the algorithm to perform the correction and eliminate false positives (for more details see 62). The minimal clusters surviving the correction were 351 cubic mm (see Table 1).

**Table 1:** fMRI results.

	**MNI coordinate**	**cluster size (mm^^3^)**	**z-value max (mean)**
**(a) Group x Emotion Interaction**			
right superior temporal gyrus	63 -15 10	5130	5.20 (3.65)
left superior temporal gyrus	-54 -33 16	2403	4.19 (3.43)
left calcarine gyrus	0 -87 10	1512	-3.89 (-3.38)
**(b) Joy > Fear z-map for the Sparse Temporal Group**			
left superior temporal gyrus	-60 -18 4	10503	6.40 (4.01)
right superior temporal gyrus	60 -15 7	8640	5.47 (3.77)
**(c) Joy > Neutral z-map for the Sparse Temporal Group**			
left inferior frontal gyrus (p. triangularis)	-42 21 1	351	5.15 (3.72)
**(d) Fear > Neutral z-map for the Sparse Temporal Group**			
right superior temporal gyrus	60 -18 10	2754	-5.03 (-3.65)
left superior temporal gyrus	-60 -18 4	2295	-4.60 (-3.59)
**(e) Joy > Fear z-map for the Continuous Temporal Group**			
calcarine sulcus	6 -99 13	783	4.46 (3.64)
**(f) Joy > Neutral z-map for the Continuous Temporal Group**			
right medial superior frontal gyrus (BA 9)	6 42 49	351	3.56 (3.29)
right superior temporal gyrus	57 -30 10	1215	4.20 (3.49)
**(g) Conjunction between "Group x Emotion Interaction" and "Joy > Fear z-map for the Sparse Temporal Group"**			
left superior temporal gyrus	-60 -21 4	1971	na
right superior temporal gyrus	45 -30 16	3024	na
right superior temporal gyrus	57 -30 19	81	na
right Heschl's gyrus	39 -27 16	27	na
**(h) Conjunction between "Group x Emotion Interaction" and "Fear > Neutral z-map for the Sparse Temporal Group"**			
right insular lobe	48 -9 4	1323	na
left superior temporal gyrus	-60 -21 7	270	na
**(i) Conjunction between "Group x Emotion Interaction" and "Joy > Fear z-map for the Continuous Temporal Group"**			
left calcarine sulcus	0 -93 13	54	na
**(j) Conjunction between "Group x Emotion Interaction" and "Joy > Neutral z-map for the Continuous Temporal Group"**			
right superior temporal gyrus	57 -30 19	432	na

(a) Results of general linear model interaction between scanning group and emotion (fear, neutral, joy), corrected for multiple comparisons (p<0.05). (b-f) Results of post-hoc comparisons between emotion conditions, performed separately for each scanning group. No difference was observed for the contrast Fear>Neutral in the continuous scanning group. (g-j) Results of conjunction between interaction effect z-map and z-maps from post-hoc comparisons.

## Results

### Behavioral Data

**Figure 2:**
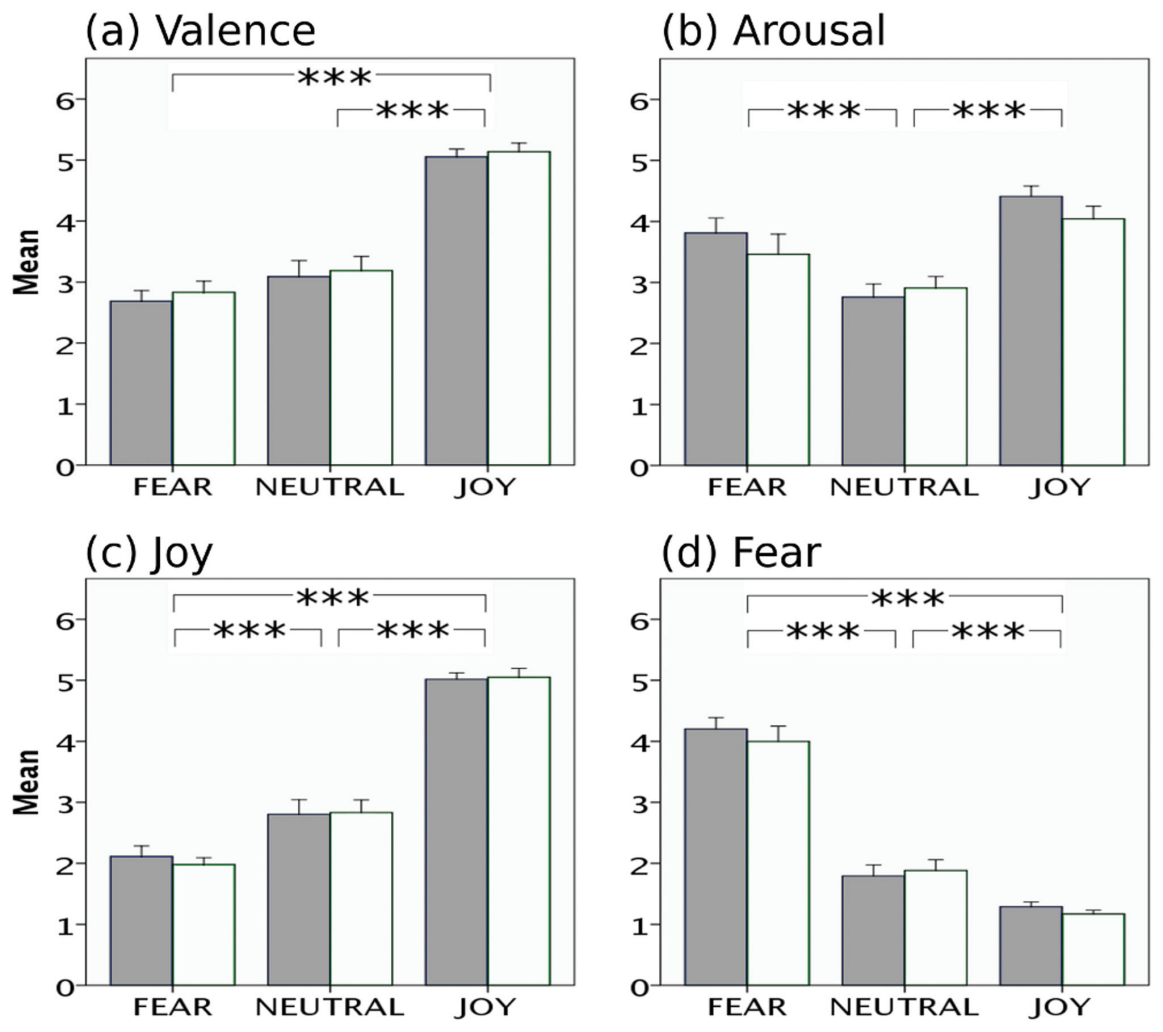
Behavioral ratings of participants on the four emotion scales used. (a) valence, (b) arousal, (c) joy, and (d) fear. Ratings are depicted separately for each stimulus category (fear, neutral, joy). White bars represent the sparse scanning group and grey bars represent the continuous scanning group. Note that there were no significant differences in ratings between the two groups. Overall, joy stimuli were rated as more pleasant than fear and neutral ones (valence/pleasantness ratings of fear and neutral did not differ). Arousal ratings of joy and fear stimuli did not differ (and both joy and fear stimuli were rated as more arousing than neutral stimuli).

Behavioral data are summarized in Figure 2 and Tables S1 and S2. All reported results were corrected for multiple comparisons. Valence (pleasantness) ratings were lower for fear than for joy stimuli (t(33) = 14.50, p < 0.0001), higher for joy than for neutral stimuli (t(33) = 10.68, p < 0.0001), but did not differ significantly between neutral and fear stimuli (t(33) = 1.82, P = 0.077).

 Arousal ratings were lowest for neutral stimuli, and highest for joy stimuli, with ratings for fear stimuli being in between. Arousal ratings differed significantly between fear and neutral stimuli (t(33) = 3.89, p < 0.0001), fear and joy stimuli (t(33) = 3.76, p < 0.0001), and between joy and neutral stimuli (t(33) = 9.14, p < 0.0001). 

 Joy ratings were lowest for fear stimuli, and highest for joy stimuli, with ratings for neutral stimuli being in between. Joy ratings differed significantly between fear and neutral stimuli (t(33) = 5.63, p < 0.0001), fear and joy stimuli (t(33) = 21.49, p < 0.0001), and between joy and neutral stimuli (t(33) = 12.72, p < 0.0001). 

 Correspondingly, fear ratings were highest for fear stimuli, lowest for joy stimuli, with ratings for neutral stimuli being in between. Fear ratings differed significantly between fear and neutral stimuli (t(33) = 10.80, p < 0.0001), fear and joy stimuli (t(33) = 18.28, p < 0.0001), and between joy and neutral stimuli (t(33) = 5.34, p < 0.0001). Independent samples t-tests showed that the ratings given to the stimuli belonging to each emotion condition did not differ between the two scanning groups (p > 0.15). 

 To test whether interaction effects between scanner noise and emotion are observable on the behavioral level, a fixed effects ANOVA item analysis was performed on the average ratings of the stimulus set. The results revealed significant interaction effects between scanner noise and emotion reflected on the average ratings of arousal F(2, 42) = 10.34, p < 0.001 and fearfulness F(2, 42) = 4.11, p < 0.05 but not on the average ratings of valence and joy (p > 0.05) (see also Figure 2). Post-hoc t-tests showed that joy and fear stimuli were rated as more arousing, and more fear-evoking by the continuous group, whereas neutral stimuli were rated as more arousing and more fear-evoking by the sparse group (p < 0.05; corrected for multiple comparisons – for detailed descriptive statistics see Table S2). 

 Average familiarity ratings did not differ significantly between joy and fear stimuli, fear and neutral stimuli, nor between joy and neutral stimuli (p > 0.20). The average ratings of familiarity did not differ between the two scanning groups for the joy stimuli, nor for the neutral stimuli or the fear stimuli (p > 0.25). 

 Each participant’s valence, arousal, and familiarity ratings were used in the fMRI data analysis as regressors of no interest (see Data Analysis). Therefore, variance related to these variables (valence, arousal, and familiarity) did not contribute to the fMRI results presented in the following. 

### fMRI data

**Figure 3:**
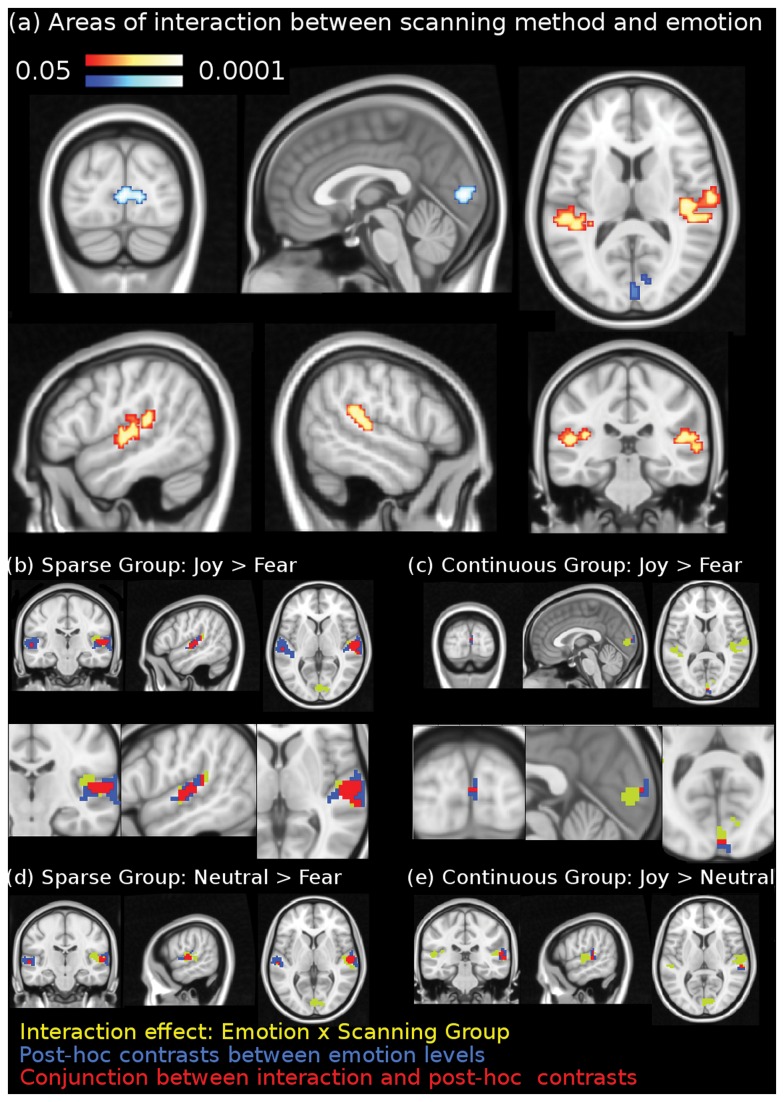
fMRI results. (a) Results of general linear model interaction between scanning group and emotional expression of stimuli (fear, neutral, joy), corrected for multiple comparisons (p < 0.05). The red scale marks clusters where the activity correlated with increases along the fear-joy emotion dimension to a greater extent for the sparse scanning group and the blue scale marks the cluster where the activity correlated with increases along the fear-joy emotion dimension to a greater extent for the continuous scanning group. (b) Conjunction between interaction effects and significant clusters of joy>fear for the sparse scanning group. (c) Conjunction between interaction effects and significant clusters of joy>fear for the continuous scanning group. (d) Conjunction between interaction effects and significant clusters of neutral>fear for the sparse scanning group. (e) Conjunction between interaction effects and significant clusters of joy>neutral for the continuous scanning group. Yellow color marks regions significant only in the z-map of the emotion by scanning group interaction. Blue color marks regions significant only in the contrast between two emotion conditions for a particular scanning group (e.g. joy>fear for the sparse scanning group; see sub-headers). Red color marks regions significant in a conjunction between the interaction effects and the z-map from a specific contrast between two emotion conditions for a particular scanning group (e.g. joy>fear for the sparse scanning group; see sub-headers).

The statistical parametric maps of the interaction Group x Emotion (corrected for multiple comparisons, p < 0.05) revealed significant interaction effects in the auditory cortex bilaterally, and in the visual cortex medially centered around the calcarine sulcus (see Table 1-a and Figure 3-a). The interaction in the auditory cortex covered auditory core, belt, and parabelt regions bilaterally, extending in the right hemisphere into the retro-insular cortex. This interaction effect was due to the effect of emotion being stronger in the sparse temporal scanning group than in the continuous scanning group. The opposite was observed in the visual cortex in the left calcarine sulcus; the peak voxel was located with 70% probability in area 17, according to probabilistic brain maps [63], where the effect of emotion was significantly stronger for the continuous scanning group. 

 To clarify the nature of these interactions, post-hoc comparisons between the three emotion conditions were performed for each scanning group separately. Results are summarized in Table 1(b-f). During continuous but not during sparse temporal scanning, fMRI activity differences between joy and fear were significant in the calcarine sulcus and greater during joy. During continuous but not during sparse temporal scanning, fMRI activity differences between joy and neutral were significant in the left auditory cortex and greater during joy. During sparse but not during continuous scanning, fMRI activity differences between joy and fear were significant in bilateral auditory cortex and greater during joy. Activity differences between fear and neutral were also significant in bilateral auditory cortex, only during sparse temporal scanning, and greater during fear. Figure 3(b-e) and Table 1(g-j) show conjunction results between the z-map of the interaction effect and the significant clusters from each post-hoc comparison. 

 The interaction effect localized in the auditory cortex was related to affective processes, rather than reflecting any difference between the two groups in low-level, purely perceptual, psychoacoustic processing. Inspection of the z−maps calculated for each of the ten psychoacoustic regressors that differed between stimulus categories showed that none of these psychoacoustic factors interacted with scanner noise, neither within, nor in the vicinity of the auditory cortex. 

 A methodological matter relevant to the results, regards the difference in the number of statistical measurements obtained for each of the scanning groups. In comparison to the continuous scanning group, there were three times fewer volumes acquired for the sparse scanning group which could result in decreased statistical power and lead to artifacts. However, the number of observations are sufficient in estimating the necessary statistics for both groups and the fact that z-values in the auditory cortex are larger for the sparse group indicates that undersampling is not the reason the presented results were observed. Moreover, the findings observed in the calcarine sulcus consolidate related findings in the existing literature [26,34], suggesting that all reported results reflect true interaction effects. This was confirmed by repeating the analysis using an equal number of observations for both groups. The right auditory cortex and visual cortex remained areas of significant interaction effects in that analysis, although these effects were not as well-localized in primary auditory and visual cortices as in the previous analysis (probably due to the decrease of statistical power associated with the use of fewer observations for the continuous group).

## Discussion

Interaction effects between emotion and scanning group were observed in the auditory and visual cortices. The interaction shows that auditory cortex activity correlates with increases towards the joy end of a “fear-joy emotion dimension”, to a greater extent during sparse than during continuous scanning. Significant increases in auditory cortex activity were observed between all emotion levels (i.e. joy vs fear, joy vs neutral, joy vs fear), only in the sparse scanning group. The interaction also shows that visual cortex activity correlates with increases towards the joy end of the fear-joy emotion dimension, to a greater extent for the continuous group and that this is mostly due to the significant increase of visual cortex activity during joy, compared to fear, in the continuous group. These results are plausible and most likely to reflect interference by scanner noise on affective neural functioning. 

 The conjunction analysis shows that parts of the auditory cortex, where significant interaction effects between scanner noise and emotion occur, bilaterally, are also regions of significant difference of activity between joy and fear as well as between neutral and fear music but not between joy and neutral music. This is only true for the sparse scanning group. In the continuous group, the opposite was observed. Part of the right auditory cortex, where a significant interaction effect between scanner noise and emotion was observed, was also significantly differing in activity only between joy and neutral music. This suggests that the presence of increased scanner noise modified the perception of the emotional qualities of the musical stimuli, on the behavioural level as evidenced by the behavioural data, as well as on the subtler level of the produced brain response, by modifying the observed relative differences between the three experimental conditions. It seems that the level of scanner noise can interact with affective neural processes by influencing the magnitude of the differential response between joy and fear, as well as between these two emotions in relation to the neutral control condition. The conjunction analysis is also demonstrating a similar observation in the visual cortex, where part of the region that is the location of the significant interaction effect between scanner noise and emotion is also an area of significant difference in activity between joy and fear, but only for the continuous group. 

That is, in the sparse group, part of the locus of the interaction effect in the auditory cortex is differing significantly in activity between joy and fear, whereas in the continuous group this difference is present in the visual cortex instead. This is likely to be due to a recruitment of emotion-processing capacities of the visual cortex in face of the desensitizing effect of increased scanner noise on the emotion-discriminative capacities of the auditory cortex. Moreover, part of the locus of the interaction effect is differing significantly in activity from neutral, during fear for the sparse group, and during joy for the continuous group. This observation implies that the increase in scanner noise influences the way neutral music is perceived, to the extent of changing its emotional quality from being more similar to happy music, in the sparse condition, to being more similar to fear music, in the continuous scanning condition. 

 The auditory cortex plays an important role in affective processing of acoustic stimuli. Differences in activity of auditory cortex between the stimulus categories were not due to psychoacoustic differences between the stimuli because these had been thoroughly controlled for during both the processes of stimulus selection/design and fMRI data analysis. Moreover, no interactions were observed in the auditory cortex between scanning group and any psychoacoustic factor. Increases in auditory cortex activity that are not due to psychoacoustic differences in the stimuli may reflect increases in the level of acoustical analysis performed and the detail of mental representations formed following increases in voluntary attention [64]. Results from several previous studies using the same or similar stimuli corroborate this view [37,65]. The interaction effect between scanner noise and emotion, observed in the auditory cortex, probably reflects that the scanner noise interferes with neural processes related to attention and detailed acoustical feature analysis, by means of its unpleasant, masking and distracting contribution to the overall acoustic percept. The continuous scanning group was exposed to more scanner noise, which blurred the perceptual borders between emotion levels. This interpretation is in line with all previous research on the effects of scanner noise on auditory cortex activity (for reviews see 11,14) and implies that increases of scanner noise impair the ability to distinguish affective differences in acoustic stimuli. The identified cluster of activity centered in the auditory cortex also covered part of the insula which is involved, amongst other processes and functions, in affective functions during cognitively demanding tasks and in emotion induction by recall and imagery [35]. The latter insular function provides a functional link with the visual cortex, additionally to well-established anatomical and functional connections between auditory and visual cortices [66]. 

 The visual cortex also plays an important role in emotion processing, though primarily of visual stimuli. Emotion induction by visual stimuli leads to increases of activity mainly in the occipital cortex and the amygdala [35]. Particularly areas of the calcarine fissure have been reported to be more responsive, in terms of activated cluster size, to emotional compared to neutral stimuli from the International Affective Picture System [34,67]. These findings are further corroborated by a reported violation of a linear response during playback of recordings of fMRI scanner noise [26]. This violation was found in a positron emission tomography study that documented a strong negative correlation between activity in the anterior calcarine cortex and accuracy in a mental imagery task. The observed correlation was present only in the absence of scanner noise. Visual imagery is considered as one basic emotion-evoking principle during music listening [68] and anatomical studies indicate that auditory core, belt and parabelt regions project to V1 and V2 of the visual cortex, and that neurons in V2 project back into these auditory regions [69]. Note that the eyes-closed requirement of the experimental task used in the present study was motivated by evidence suggesting that affective activity is enhanced when the eyes are closed [70], a condition that practically minimizes any vision-specific sensory contributions to visual cortex activity. Evidence suggesting that the occipital visual cortex is also involved in spatial hearing, in people with normal sight, have also been observed during several different auditory tasks (for details see 71). Based on the existing literature on the topic, the most plausible interpretation of the findings related to visual cortex activity is that they reflect interference due to scanner noise on the mental imagery constituents of affective processing. In the context of the experimental task used for the present study, which required extensive emotional ratings after each stimulus, the continuous scanner noise may have forced the participants belonging to the continuous scanning group to rely more on visualization for perceiving the emotion differences in the music. That is, some of the affective functionality of the visual cortex may have been recruited in the continuous group to compensate for the diminished activity in the auditory cortex. A complementary perspective, in line with previous findings, suggests that since the activity in the calcarine cortex correlates negatively with concentration and performance [26], the continuous scanning group was less concentrated due to the distracting effects of the greater amount of scanner noise it was exposed to. 

 No interaction effects were observed in any limbic structures, commonly associated with emotions, such as the amygdala, ventral striatum and hippocampal formation. There are two factors that might be contributing to this. Firstly, as previously demonstrated [39], activity in these structures is sensitive to scanner noise and becomes observable only under optimized conditions featuring minimized scanner noise. Secondly, sensitivity to changes of activity in limbic structures improves with increasing field strength [72]. The present study was conducted with a field strength of 1.5 Tesla and a standard 12-channel head coil, which may not have allowed for the detection of interaction effects in limbic structures using the particular stimulus set. 

 The observed data suggest scanner noise to be a more significant confound for fMRI research than previously believed. In addition to scanner noise effects on the responsiveness of the auditory cortex, the presented data show that scanner noise interacts with affective processes. Thus, scanner noise affects a wide range of basic aspects of brain functioning: from sensory components [19,20,26,28,29], to cognitive domains such as attention [30] and memory [27,30], as well as motor function [19] and emotion, as suggested previously [39] and evidenced by the present study. 

 Such findings were expected because it has been long known that exposure to noise is associated with annoyance reactions [73], hypertension, cardiovascular disease, catecholamine secretion, psychological symptoms, impaired reading comprehension, impaired memory skills (for a review see 74) and possibly prevalence of psychiatric disorder [75]. Extensions of the presented work would caution one to the implications that long-lasting exposure to similar types of noise could possibly have on the neural functioning of a population at large (see also 76). 

 Minimizing noise exposure during fMRI scanning is of major importance, especially in obtaining accurate readings of affective neural processes. To this end, many modifications to the usual scanning implementation have been proposed, including software optimization, active noise-reduction technologies and hardware enhancements. 

 With regards to software optimization, modified acquisition sequences such as Stimulated Echo Acquisition Mode (STEAM) [1,77-79]; Simultaneous Multislice Excitation (SIMEX) combined with Fast Low Angle Shot (FLASH) or spiral imaging [1,28,29]; Functional Burst Imaging (FBI) [80]; Sensitivity-Encoded Echo-Planar Imaging (SENSE-EPI) [81]; Interleaved Silent Steady State imaging (ISSS) [82]; Sweep Imaging with Fourier Transformation (SWIFT) [83]; and others varying the projection reconstruction method while optimizing gradient pulsing [1] or minimizing the empty space in the field of view and volume acquisition time through interleaved spiral trajectory k-space imaging [84] are representative of progress in scanner noise reduction. 

 Monitoring scanner noise with microphones while simultaneously generating and adding the inverse soundwave to the output of the stimulus-presentation headphones is an application instance of a technology known as Active Noise Cancellation [85-87]. An extension of the theory behind active noise cancellation has led to Active Structural Acoustic Control that uses panels featuring vibro-acoustic sensors and active actuators to introduce anti-vibrations on solid materials [88]. 

 With regards to hardware enhancements, using vacuum-enclosed heavy gradient coils, heavy mounting to the floor to maximize vibrational absorption [1,89] and implementing active Lorentz force balancing applied to gradient coil design [90-92] have been suggested to decrease scanner noise [1]. Hardware customizations implemented in “bench-top prototype two-coil systems” [90], research scanners featuring high noise reduction and commercial “quiet MR-systems” [8,89,93], make scanning less noisy and the cost estimates are reasonable in comparison to the cost of the fMRI experiments that are being conducted on more than 20000 fMRI scanners worldwide [94] producing results that are susceptible to noise interference and should be treated with caution. Considering that most of these solutions have been in place for over a decade and that there has been little development in this field since that time, it is in the interest of valid scientific practice to treat the elimination of scanner noise as a pressing issue of high priority. 

## Conclusion

It has been demonstrated experimentally that scanner noise interactions with affective neural processes can occur in the auditory, retro-insular and visual cortices during emotional music listening. Such interactions are likely to reflect involuntary changes in levels of attendance to auditory stimuli and to be related to processes of attention and visual imagery. 

## Supporting Information

Table S1 **Behavioral Ratings.** Descriptive statistics of behavioral data (mean, with standard deviation in parentheses). For statistical tests see main text.
(DOC)

Table S2 **Detailed Statistics.** Descriptive statistics of behavioral ratings for each scanning group and emotion category (mean, with standard deviation in parentheses). For statistical tests see main text.
(DOC)

Table S3 **List of fear and joy stimuli.**
(DOC)
