# Supplementary material for: fMRI Scanner Noise Interaction with Affective Neural Processes
Source: PLoS One. 2013 Nov 18;8(11):e80564. doi: 10.1371/journal.pone.0080564 (PMC3832369; doi:10.1371/journal.pone.0080564)
Supplement: Table S1 — Behavioral Ratings. Descriptive statistics of behavioral data (mean, with standard deviation in parentheses). For statistical tests see main text. (DOC) [file pone.0080564.s001.doc]

**Table S1: Behavioral Ratings.**

|  | **Fear** | **Neutral** | **Joy** |
| --- | --- | --- | --- |
| **Valence** | 2.43 (0.20) | 2.69 (0.48) | 4.84 (0.21) |
| **Arousal** | 3.97 (0.21) | 3.03 (0.22) | 4.05 (0.21) |
| **Joyfulness** | 1.62 (0.16) | 2.40 (0.29) | 4.83 (0.42) |
| **Fearfulness** | 4.02 (0.23) | 2.22 (0.30) | 1.31 (0.17) |
| **Familiarity** | 1.44 (0.11) | 1.17 (0.10) | 2.01 (0.42) |

Descriptive statistics of behavioral data (mean, with standard deviation in parentheses). For statistical tests see main text.
