# Supplementary material for: fMRI Scanner Noise Interaction with Affective Neural Processes
Source: PLoS One. 2013 Nov 18;8(11):e80564. doi: 10.1371/journal.pone.0080564 (PMC3832369; doi:10.1371/journal.pone.0080564)
Supplement: Table S2 — Detailed Statistics. Descriptive statistics of behavioral ratings for each scanning group and emotion category (mean, with standard deviation in parentheses). For statistical tests see main text. (DOC) [file pone.0080564.s002.doc]

**Table S2: Detailed Statistics.**

|  | **Joy** | | **Neutral** | | **Fear** | |
| --- | --- | --- | --- | --- | --- | --- |
|  | **Sparse**  **mean (SD)** | **Continuous**  **mean (SD)** | **Sparse**  **mean (SD)** | **Continuous**  **mean (SD)** | **Sparse**  **mean (SD)** | **Continuous**  **mean (SD)** |
| **Valence** | 5.14 (0.23) | 5.06 (0.19) | 3.19 (0.24) | 3.09 (0.18) | 2.83 (0.18) | 2.69 (0.20) |
| **Arousal** | 4.04 (0.25) | 4.41 (0.26) | 2.91 (0.23) | 2.76 (0.18) | 3.46 (0.15) | 3.81 (0.34) |
| **Joyfulness** | 5.05 (0.31) | 5.02 (0.36) | 2.83 (0.23) | 2.80 (0.21) | 1.98 (0.16) | 2.11 (0.19) |
| **Fearfulness** | 1.17 (0.12) | 1.29 (0.22) | 1.88 (0.20) | 1.79 (0.20) | 4.00 (0.20) | 4.20 (0.31) |

Descriptive statistics of behavioral ratings for each scanning group and emotion category (mean, with standard deviation in parentheses). For statistical tests see main text.
