# Supplementary material for: fMRI Scanner Noise Interaction with Affective Neural Processes
Source: PLoS One. 2013 Nov 18;8(11):e80564. doi: 10.1371/journal.pone.0080564 (PMC3832369; doi:10.1371/journal.pone.0080564)
Supplement: Table S3 — List of fear and joy stimuli. (DOC) [file pone.0080564.s003.doc]

**Table S3:** List of fear and joy stimuli.

| **Joy-evoking** | | |
| --- | --- | --- |
| **Composer / artist** | **Title** | **ASIN Nr.** |
| Joel Francisco Perri | El Canto de Mi Antara | B002BEXEHO |
| Craobh Rua | The Lucky Penny | B000003NHN |
| Scotch Mist | Shetland Tune | n/a |
| Alfredo de Angelis | Pregonera | B001P5LDTQ |
| Orchestra Paraschiv Oprea | Batuta de la Adancata | B00000DTI6 |
| Louis Armstrong | St. Louis Blues | B006CBVRJY |
| Niccoló Paganini | Violin Concerto No. 1, 3rd movement | B000001GHC |
| Jonathan Richman | Egyptian Reggae | B00580D1IU |
| **Fear-evoking** | | |
| **Composer / artist** | **Title** | **ASIN Nr.** |
| Danny Elfman | The Killing | B00000JC9R |
| Michael Giacchino | Monsters Are Such Interesting People (from: Lost) | B000EHSVDM |
| Michael Giacchino | Just Another Day on the Beach (from: Lost) | B000I2IQ9M |
| Michael Giacchino | Charlie’s Dream (from: Lost) | B000I2IQ9M |
| Takeshi Miura et al. | Pulsating Right Arm (from: Biohazard Code) | B00005HWMB |
| Seiko Kobuchi | Boss Battle (from: Biohazard Zero) | n/a |
| Masami Ueda & Saori Maeda | Cold Sweat (from: Biohazard 3) | B000058A7Y |
| Akihiko Matsumoto et al. | Freezer Burn (from: Resident Evil Outbreak) | B00019257G |
